# Human perception and biosignal-based identification of posed and spontaneous smiles

**DOI:** 10.1371/journal.pone.0226328

**Published:** 2019-12-12

**Authors:** Monica Perusquía-Hernández, Saho Ayabe-Kanamura, Kenji Suzuki

**Affiliations:** 1 Communication Science Laboratories, NTT, Atsugi, Kanagawa, Japan; 2 Artificial Intelligence Laboratory, University of Tsukuba, Tsukuba, Ibaraki, Japan; 3 Faculty of Human Sciences, University of Tsukuba, Tsukuba, Ibaraki, Japan; Birkbeck University of London, UNITED KINGDOM

## Abstract

Facial expressions are behavioural cues that represent an affective state. Because of this, they are an unobtrusive alternative to affective self-report. The perceptual identification of facial expressions can be performed automatically with technological assistance. Once the facial expressions have been identified, the interpretation is usually left to a field expert. However, facial expressions do not always represent the felt affect; they can also be a communication tool. Therefore, facial expression measurements are prone to the same biases as self-report. Hence, the automatic measurement of human affect should also make inferences on the nature of the facial expressions instead of describing facial movements only. We present two experiments designed to assess whether such automated inferential judgment could be advantageous. In particular, we investigated the differences between posed and spontaneous smiles. The aim of the first experiment was to elicit both types of expressions. In contrast to other studies, the temporal dynamics of the elicited posed expression were not constrained by the eliciting instruction. Electromyography (EMG) was used to automatically discriminate between them. Spontaneous smiles were found to differ from posed smiles in magnitude, onset time, and onset and offset speed independently of the producer’s ethnicity. Agreement between the expression type and EMG-based automatic detection reached 94% accuracy. Finally, measurements of the agreement between human video coders showed that although agreement on perceptual labels is fairly good, the agreement worsens with inferential labels. A second experiment confirmed that a layperson’s accuracy as regards distinguishing posed from spontaneous smiles is poor. Therefore, the automatic identification of inferential labels would be beneficial in terms of affective assessments and further research on this topic.

## Introduction

Assessing affective experience is relevant in many application domains. These range from tracking therapy results and augmented feedback for people with physical or mental impairments [1, 2]; user and customer experience mapping [3–5]; to human-robot interaction [6]. However, measuring affect in a continuous, accurate, and reliable manner is still a challenging task. Embodied behaviour, such as facial movement and body movement, has long been considered an alternative to measuring emotion [7, 8]. Early theories on emotion processes suggested that changes in our body states create the subjective feeling of an emotion [9, 10]. In this view, humans share a common pattern of emotional expression via facial expressions as a result of natural selection [11]. The emotions conveyed by the face can be therefore regarded as universal [9]. Within the framework of the Basic Emotion Theory (BET), several prototypical facial expressions are believed to be hardwired and mapped to a specific felt emotion [12, 13]. Evidence from congenitally blind people who smile when happy, or display signs of sadness, supports this view [14]. However, facial expressions can also be produced voluntarily, and they are often used to provide misleading information about the producer’s emotional state [15–19]. Moreover, facial expressions can be used as a social signalling tool with several functions. For example, smiles are not only an expression of joy. They have also been shown to denote reward, affiliation, and dominance during reward behaviour, social bonding, and when negotiating a hierarchy [20]. In another study, smiles were found to signal both amusement and contempt. Moreover, undesired signalling is also avoided by deliberately dampening these smiles [21].

Recently, facial expressions of emotion have been interpreted in other ways due to the lack of coherence between a facial expression and the experienced emotion. For example, the behavioural ecology view (BECV) of facial displays rethinks facial expressions of emotion as contingent actions for social negotiation [22]. In other words, the BECV understands a facial expression of emotion in terms of what observers understand of it, and not in terms of the inner affective state that caused it. Furthermore, other models fall in between the BET and the BECV extremes. For example, the Componential Processing Model (CPM) [23] of emotion appraisal suggests that both internal and social (i.e, environmental) cues affect how people respond. Within the framework of the Facial Action Coding System (FACS) [24], it has been observed that some Facial Action Units (AU) are reliable and others versatile. Reliable AUs appear to be more difficult to control than versatile AUs. Moreover, the activation of reliable AUs has a stronger effect on the meaning of the perceived facial expression [25].

The FACS has been widely used to annotate facial expressions. FACS rating involves identifying muscular movements, or Action Descriptors, for movements involving multiple muscles on a frame-by-frame basis from their onset to offset. Peak contraction is then noted on a scale ranging from one to three or one to five, depending on the coders. From these AU configurations, inferences can be made by experts in the frame of different theories. The co-occurrence of emotional states and AUs can be analysed in the frame of the BET; the influence of contextual cues on the AU presence and absence can be analysed in the frame of the BECV; and AU combinations that take into account appraisals made in a sequential and iterative process can be analysed in the framework of the CPM. This is the main advantage of the FACS: the observable AUs that convey a message are described and counted before making more subjective inferences about their meaning. As described in the FACS Investigator’s Guide [26], the FACS methodology only measures the sign vehicles that convey a message. In other words, the target behaviours are described and counted. These behaviours include counting how many times a muscle moves or registering the duration of the movement between onset and offset. Henceforth we refer to the generated labels as a “perceptual ground truth” because this type of video rating relies only on perceptual movements. After a perceptual ground truth has been established, those labels are used to make judgments about the given message. In these message judgments, inferences underlying the behaviour are made. Thus, henceforth we refer to the messages inferred from the perceptual cues as “inferential ground truth.”

Since video rating according to the FACS relies on descriptions of behaviour units, it is considered to be a perceptual ground truth. However, it assumes that the human observer is trained to reliably recognize and name specific AUs. Hence, the inference level is a step higher than simply rating facial movement alone as it relies on an agreed label set. Furthermore, facial expressions are usually labelled by third-person coders under the assumption that they can have a more objective perception of the facial expression than those experiencing the emotion. For example, the person displaying the emotion might be more biased towards describing what they remember than what they see [27]. Moreover, facial expression labelling are tedious and time-consuming. Also, it is difficult for two human coders to completely agree on the labelled behaviour. The perception of facial movements has some error associated with it. This error is caused by perceptual limitations, or by conceptual mismatches as regards label meanings. It is important to notice that accuracy and error in the context of human rating refer to disagreement with respect to the relationship between the observed behaviour and the target label. Different coders might disagree on the occurrence of the labelled behaviour. Nevertheless, human rating is still considered the ground truth in most studies. When the task is solely to identify and count visible behaviours, this seems to be the most appropriate ground truth label. However, for some applications identifying the presence or absence of affective cues and counting them is insufficient. Since humans excel at controlling their own facial movements, it is easy for a person to feign an emotion [17]. Hence, using perceivable facial expressions as a measure of affect is prone to biases and manipulations analogous to those found with self-report. Examples are the demand characteristics bias, or the tendency of participants to play a good role and respond according to their guess regarding their expected answer [28, 29]; and the Social Desirability Bias, or the tendency of people to self-report inaccurately to present themselves in the best possible light [30].

Because of the voluntary production of facial expressions and the confounding that this entails, our first research question is **to what extent are third-person video-rated ground truths for facial expression spontaneity accurate? If the judgment of a third person is inaccurate, more importance should be given to situations where facial expressions were produced than to labelled ground truths only (H1)**. These situations include both (1) the experimental design used to elicit the facial expressions, or possible motives to endogenously emit one; and (2) more ecologically valid contexts in which the facial expression occurred. For example, it would be more credible to consider a smile as spontaneous during game play, than when greeting our boss on Monday morning.

### Posed and spontaneous facial expressions of emotion

As previously described, facial expressions of emotion can be produced voluntarily. They can be used as a communication tool to convey an emotion, even if it is not felt. In this case, the producer is in control of the affective state or message to be transmitted. Therefore, facial expressions of emotion may be emitted (i.e., they have an endogenous source) or elicited (i.e., they have an exogenous source) [31]. Previous research has named these two types of expressions posed, deliberate, or voluntary; and spontaneous or genuine, respectively [32]. Nevertheless, there is no one-to-one mapping between posed and spontaneous categories. They often appear mixed with each other. For example, people can produce endogenous smiles that are spontaneous when thinking of past events or making an association with something funny.

Previous work has suggested that deliberate and spontaneous facial expressions of emotion have different characteristics. They often involve different facial muscles [17]; their temporal dynamics are different [32]; and they are even mediated by distinct neural pathways [17]. For example, consider the case of a smile. A smile usually conveys a positive affect state, but this state can be felt or expressed out of politeness [33, 34]. Several differences have been found between posed and spontaneous smiles. Arguably, the main difference is the activation of the *orbicularis oculi* muscle (Duchenne marker) during spontaneous or Duchenne smiles only [13, 32, 35, 36]. However, other studies have found that this muscle is activated in both types of smiles [37–39]. Moreover, the Duchenne marker might also signal smile intensity instead of smile authenticity [38, 40, 41]. Furthermore, posed smiles tend to have a larger amplitude [37, 42, 43]; different onset and offset speed and duration [37, 44, 45]; and a different number of peaks [45]. Additionally, spontaneous smiles tend to last longer than posed ones [37, 43], and have a fast and smooth onset [42]; with apex coordination, in which muscle contractions in different parts of the face peak at the same time [17]. Dynamic aspects of facial expressions have indeed been argued as critical to facial expression recognition accuracy as perceived by humans. This is especially true for subtle expressions, and when static information is of low quality [46–48]. Therefore, dynamic information is a promising resource for inferring and communicating meaning from facial expressions of emotion.

Understanding others’ facial expressions is a critical social ability. Therefore, human perception of posed and spontaneous facial expressions has been extensively researched. The message transmitted by each facial expression is as important as the actual context in which they were emitted or elicited [49], as they transmit both biologically basic and socially specific messages [50]. Nevertheless, several studies have found that humans have only a moderate ability to distinguish between these two types of expressions. In the case of pain, the human ability to distinguish between real and fake expressions is no better than chance [51]. Similarly, the accuracy with which human judges can distinguish between spontaneous and both improvised and rehearsed posed expressions of surprise is around 50% [52]. Other studies have shown that human judges can distinguish between posed and spontaneous displays of emotion for amusement, surprise, disgust, and sadness. However, this sensitivity depends on the dynamic presentation of the facial display [48]. Furthermore, only moderate accuracy was achieved by human judges when distinguishing between different types of acted smiles, even though the prototypical characteristics of each smile type might have been enhanced by the actors [47]. Given the above, technology with high temporal resolution might prove advantageous in terms of automatic recognition beyond human perception.

### Automatic identification of facial expressions of emotion

Automatic identification of facial expressions is a tool that has gained popularity in recent years. Its main advantage is its reliability, or the extent to which the results can be reproduced under the same conditions. Current automatic identification algorithms take into consideration only physical factors and do not conflate them with prior beliefs. Moreover, most sensors have higher temporal resolution than that of self-report. Several surveys have been conducted to summarise the different signals that can be used for technology-afforded emotion recognition [53–55]. However, most systems can only claim a high perceptual agreement between facial movement perceived by a machine and a human, without necessarily providing insights into the underlying emotion, because of the lack of congruence between facial expressions of emotion and felt affect. Hence, interpretation of the facial movement is usually left to an expert.

There are different methods of establishing the ground truth used to train these systems. These methods include using the video rating of facial expressions, self-reported labels, and most importantly, labels related to how the data was acquired. Video rating is still the most commonly used method for labelling facial expressions, and labels are still assigned by a third-person human coder. Video rating is specially suitable for cases where no ground truth information is available to perceptually label facial movement and infer its meaning. However, for inferential judgements on the facial expression meaning, we argue that **if technology can pick up spatio-temporal dynamics in a reliable and holistic manner, even if no AU labels are used, automatic identification would complement human inferential judgments about smile spontaneity (H2)**. In this case, the challenge lies in correctly inferring a person’s intention or lack of intention by distinguishing between posed and spontaneous smiles. Therefore, special attention must be paid to the methods used for acquiring the data to train such systems.

Recently, Computer Vision (CV) is the most widely-used tool for automatically identifying facial expressions or posture [32, 56]. This method has led to an identification accuracy of close to 90% [57]. Moreover, the use of spatial patterns has been shown to achieve about 90% accuracy in the task of distinguishing between posed and spontaneous smiles [58]. In particular, the publication of the UvA-NEMO database [59], including 1240 videos of spontaneous and posed smiles, has triggered a renewed interest in identifying the differences between posed and spontaneous smiles and their dynamic characteristics [32]. State-of-the-art methods have provided an identification accuracy of up to 92.90% by using dynamic features based on lip and eye landmark movements, sometimes tailored to different age groups [59]. Other algorithms using spatio-temporal features as identified by restricted Boltzmann machines have been able to achieve up to 97.34% accuracy with the UvA-NEMO database, and 86.32% with the Spontaneous vs. Posed Facial Expression (SPOS) database [60].

Besides CV-based methods, the potential of EMG to study different facial expressions has been extensively studied either by placing recording electrodes near the muscles [8, 17, 61–66], or with innovative wearable devices that do not obstruct the face [67–72]. While state-of-the-art cameras can achieve a couple of hundreds of frames per second, Electromyography (EMG) can realise 1000 samples per second, thus increasing the temporal resolution with which changes can be detected. This might be advantageous for the EMG-based identification of subtle expressions that are imperceptible visually [62]. Moreover, posed and spontaneous smiles can also be distinguished from EMG features. Surface EMG has revealed that spontaneous smiles have different magnitudes, speeds and durations than posed smiles [42, 43]. Posed and spontaneous smiles can also be distinguished by employing wearable facial distal EMG [72]. Spatial and magnitude feature analysis provided an accuracy of about 74% when distinguishing between posed and spontaneous smiles. On the other hand, the accuracy reached about 90% and the inter-individual variability was reduced by using spatio-temporal features. Therefore, both types of smiles differ as regards onset and offset times rather than magnitude.

Despite the good results claimed in previous studies, the temporal dynamics of the reported posed smiles could have been affected by the duration of the instruction given to the participants. Since posed expressions were emitted on command, their temporal dynamics might have been constrained. Therefore, further exploration is needed to confirm the differences between the temporal dynamics of posed and spontaneous expressions. **If there is a confounding of the instruction duration to pose a smile, then posed smiles emitted when following an instruction are different from posed smiles emitted with the intention of signalling enjoyment in slightly negative situations (H3)**.

A recent study, [73] compared posed and spontaneous smile detection using both CV and facial distal EMG methods to investigate whether invisible potentials were informing the distinction. The mean accuracy of a intra-individual spatial features algorithm was 88% for CV, and 99% for EMG. With intra-individual spatio-temporal features, the mean accuracy was 87% for CV, and 91% for EMG. This suggests that EMG probably has the advantage of being able to identify covert behaviour that cannot be detected visually in intra-individual models. However, the predictive analyses were used only to assess whether the differences between smile types are identifiable. Also, no detailed descriptions were provided of the smile characteristics that are important to differentiate them.

### Cultural diversity in smiling behaviour

It is important to note that most previous studies have used only Western, educated, industrialised, rich and democratic (WEIRD) samples [74] for their studies despite evidence indicating that cultural diversity influences the production and perception of smiling behaviour. Smile and laughter displays by citizens of countries with ancestral diversity are easier to decode by observers, and they smile more in response to certain stimuli than residents of countries that lack ancestral diversity [75]. Moreover, posed or polite facial expressions might differ depending on cultural background. [76] showed evidence that when posing smiles, Canadians typically show the Duchenne marker, but Gabonese do not. On the other hand, mainland Chinese participants were sensible to the Duchenne marker only when judging smiles from French-Canadians. This suggests that the marker is learned through cultural context. A follow-up study explored whether or not children used the Duchenne marker as a visual cue to distinguish between the two types of smiles. According to their results, children between 4 and 17 years old perceive medium Duchenne smiles as more authentic than equally intense medium non-Duchenne smiles, and they rely less on the intensity of the smile as they grow older [77]. Moreover, Chinese who use the eyes as a cue when interpreting the facial expression of another person are more accurate than those who use the mouth. Furthermore, those who rated themselves as caring about other people tended to be more accurate and sensitive to the Duchenne marker. Even though these articles support the hypothesis that the ability to pose a smile and to distinguish between posed and spontaneous smiles is acquired through socialisation, their definition of spontaneous or genuine smiles is based on the Duchenne marker. Nevertheless, the ability to display this marker can be learned [78]. Thus, a Duchenne smile is not necessarily spontaneous [37–39]. Despite this, many studies draw conclusions regarding the perceived spontaneity of a smile using only the Duchenne marker [79, 80], and sometimes only in static pictures [81]. Additionally, Cross-Race Effects (CRE), or Cross-Ethnicity Effects, are a well-replicated face recognition finding where people are better at recognising faces from their own ethnicity, relative to other ethnicities [28, 82]. These differences in ethnicity might also be correlated with smile spontaneity judgments. Based on the aforementioned differences, our last research questions are: **to what extent is the production of posed and spontaneous smiles influenced by the ethnicity of the producers? (H4a)** and **to what extent is the perception of posed and spontaneous smiles influenced by the ethnicity of the producers and perceivers? (H4b)**.

## This study

In this study we explore the differences between posed and spontaneous smiles from their EMG signatures, and to what extent the self-perceived spontaneity of a smile relates to an analogous third person’s judgment. In two experiments, this paper aims to:

1. establish a method for eliciting balanced quantities of spontaneous and posed smiles in controlled settings;
2. report the EMG spatio-temporal signatures of spontaneous and posed smiles that were collected without a time-constrained command;
3. to compare human and automatic identification. The main differences between this and previous work are the characteristics of the collected posed smiles; and
4. propose that the ground truth for the automatic identification of posed and spontaneous smiles ought not be a third person’s judgment, as this seldom agrees with the ground truth established by the elicitation method and self-report.

While previous research has found definite spatio-temporal characteristics for posed smiles when compared with spontaneous smiles, the gathered posed smiles were deliberate smiles “produced volitionally in response to a specific interviewer request” [37], to “pose an enjoyment smile as realistically as possible” [59], or the participants were just “asked to pose smiles” [72]. Moreover, these posed smiles were produced in the absence of any induced affect.

In experiment 1, differences between the production of posed and spontaneous smiles were outlined based on distal facial EMG for producers of both Asian and Non-Asian ethnicity. In contrast to previous studies, the emitted posed smiles were not smiles posed for the camera. They were smiles intended to show enjoyment under slightly negative circumstances, and the duration of each smile was decided entirely by the producers. By employing this approach, we hoped to have more ecologically valid smile samples to corroborate the role of the spatio-temporal features in differentiating deliberate smiles from spontaneous smiles. The obtained smiles differed in magnitude, onset time, and onset and offset speed independently of the producer’s ethnicity. Additionally, an EMG-based detection technique was implemented. Its accuracy was determined against a ground truth composed of the human rating of facial expressions, self-report, and most importantly, the experimental design used to collect the data. This algorithm aimed to make an inference on the genuineness of a smile in an holistic manner. Joint facial muscle activity was used to calculate spatio-temporal features, without focusing on individual facial movements. Furthermore, independent human coders labelled the data. Perceptual judgments on facial movement yielded a higher degree of agreement than inferential judgments on smile spontaneity. Moreover, the agreement between independent coders and the producer’s results is even lower and slightly affected by the ethnic background. Therefore, electrophysiological and behavioural signal-based solutions appear to be more reliable than inferential judgments. Experiment 2 was designed to further explore the degree to which a third-person video rater would be able to distinguish between those two expressions. The results showed that the identification accuracy for human judges is very modest, and there is a trend indicating that ethnicity mismatches might affect spontaneity judgment accuracy.

## Experiment 1: Spatio-temporal responses during spontaneous and posed smiles

### Participants

41 volunteers took part in the study (19 female, average age = 25.03 years, SD = 3.83). 25 were Asians (Japan and China), and 16 non-Asians (Brazil and Mexico). Henceforth, the participants in this experiment are called “producers”. All the participants had normal or corrected-to-normal vision. This research was approved by the Institutional Ethical Committee of the University of Tsukuba with review code 2017R176. All the producers provided written informed consent at the beginning of the experiment, and verbal consent at the beginning of each experimental block.

### Experiment design

The producers were invited to a “video rating” experiment. They were told that the goal was to rate the content of some videos and to determine how comfortable it was to wear the provided sensing wearables. They were informed that the experiment consisted of several blocks and that the instructions for each block would be provided before starting each one. If they agreed to participate, they were asked to sign an informed consent. Next, they were shown a picture of the wearables and assisted to wear them by the experimenter.

**Fig 1:**
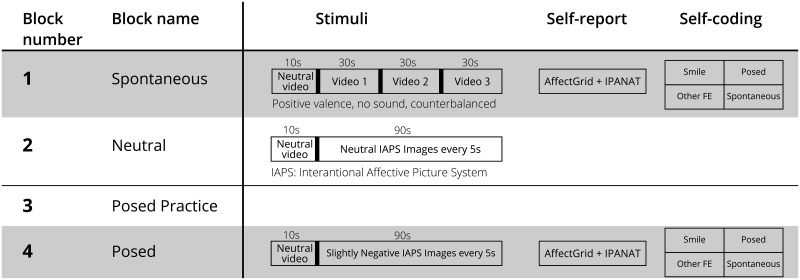
Experimental design for experiment 1. All producers went through all six experimental blocks in the same order. The first block was designed to induce positive affect and therefore smiling behaviour. The second block was designed to reset that affective valence. The third block provided an opportunity to practice a posed smile when smiling for the camera. The fourth block was designed to induce a slightly negative feeling while people were asked to smile deliberately.

The experiment consisted of four blocks (Fig 1):

1. **Spontaneous Block (S-B)**. A positive affective state was induced using humorous videos, and therefore, the emission of spontaneous smiles was promoted. Producers were asked to watch the stimuli videos and relax. During the stimuli, the experimenter retreated to a different section of the room, which was created by using a temporary divider. After the stimuli, an explanation was provided of how to answer two standardised scales assessing emotional experience, and several video preference questions. Next, producers were asked to tag any facial expressions that they had made during the S-B. They were allowed to practice with a one-minute video.
2. **Neutral Block (N)**. It aimed to convert the positive affect elicited during S-B into a neutral affect. The producers were told to watch a video and relax.
3. **Practice posed smile (P)**. The producers were asked to pose a smile (P) for the camera for approximately 5 s to check the sensors’ recordings. The purpose of this smile was two-fold. First, it served as practice for the next block. Second, it was a posed smile produced in accordance with an instruction. Next, the producers were debriefed. An explanation regarding the goal of distinguishing between posed and spontaneous smiles was provided.
4. **Posed Block (P-B)**. Producers were requested to make similar facial expressions as in the S-B. However, this time, a slightly negative video was presented instead. Their instruction was: “Please perform the smiles you video coded. This is for a contest. We are going to show the video we record to another person, who is unknown to you, and if she or he cannot guess what video you were watching, then you are a good actor. Please do your best to beat the evaluator”. Producers were simply asked to do their best, no other reward monetary or otherwise was promised. This type of smile was considered to be a re-enactment posed smile that deliberately intended to give the impression of having fun by remembering past experiences. With this, special care was taken to elicit a balanced number of facial expressions with a known ground truth and without modifying their temporal characteristics. After watching the video and performing the task, they completed the same two standardised scales assessing emotional experience as in the S-B. Demographics and control questions were included afterward. Next, they were asked to tag their own expressions as before.

All the producers completed all the experimental blocks in the same order. This was to keep the purpose of the experiment hidden during the spontaneous block. Only the stimuli videos inside the spontaneous block were counterbalanced. Given the self-report, the self-video rating, the N-B, P, and the debriefing, the time between S-B and P-B was about 30 minutes. At the end of the experiment, the producers were debriefed and thanked for their participation with a gift voucher with an approximate value of 5 USD.

### Stimuli

During the Spontaneous, Neutral, and Posed Blocks, 90 s videos were used as stimuli. Each stimulus video was preceded by a 10 s neutral video designed to establish a relaxing baseline. The videos had the following content:

- **Pre-block stimuli**. A video of raindrops falling on the camera lens was shown for 10 seconds.
- **Spontaneous Block**. Three 30 s videos were concatenated with a 1 s black transition. These were popular internet videos successfully used in previous research for eliciting positive expressions [72, 83]. They feature a baby being surprised by a simple magic trick [72]; a panda calling for the attention of a zookeeper [84]; and a cat moving rhythmically as his owner petted it [85]. These were intended to match the preferences of most of the producers. The three videos were presented in a counterbalanced order that included all six combinations.
- **Neutral Block**. The neutral block video consisted of 18 pictures from the International Affective Picture System (IAPS) [86], with likeability scores between 5.0 and 6.0, presented every 5 s, for a total of 90s. The IAPS picture reference numbers were: 1670, 1908, 2025, 2273, 5390, 5500, 7052, 7211, 7351, 7496, 7509, 8465, 2235, 2382, 2488, 7354, 7490 and 7503. Hence, the duration of the neutral video was the same as that of the spontaneous block.
- **Posed Block**. Similarly, 18 IAPS pictures with likeability scores between 4.0 and 5.0 were selected and presented every 5s for a total of 90s. The IAPS picture reference numbers were: 1505, 2130, 2272, 2309, 5120, 7013, 7234, 7290, 7487, 7590, 8010, 8475, 2101, 2770, 5970, 6800, 9472 and 9913. The images chosen for this block had a mildly unpleasant valence. For example, these images included pictures of raw fish, cyclists crossing the railway when a train is approaching, and a cow going out of control.

The dynamic motion of the stimuli in the neutral and posed blocks was restricted to several images presented sequentially rather than more dynamic videos to avoid eliciting strong facial expressions other than smiles.

### Measurements

**Fig 2:**
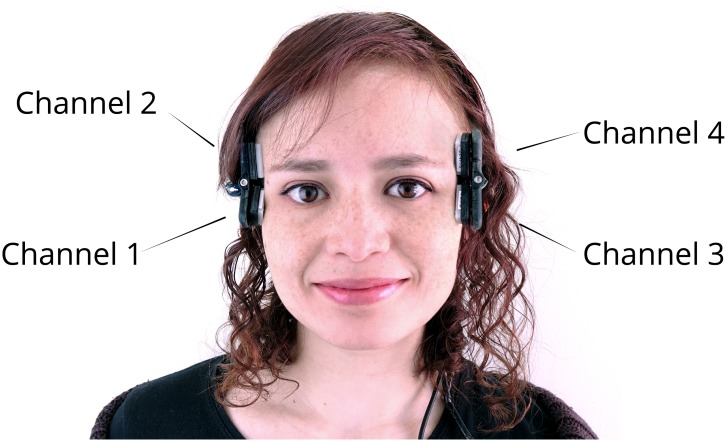
Smile reader. The EMG wearable consists of four channels located as depicted in this figure. *Written informed consent was obtained for the publication of this image*.

- **Smile-reader**. Four channels total of distal facial EMG were measured from both sides of the face using dry-active electrodes (Biolog DL4000, S&ME Inc). Fig 2 shows the EMG channel position implemented on a wearable designed to keep the electrodes in place. This placement has been shown to be able to reliably measure smiles in different situations [68–71]. By placing the electrodes on the sides of the face, facial movement remains unobstructed. EMG electrodes are traditionally pasted on top of the relevant muscle, thus interfering with natural movement. By avoiding this placement, research on spontaneous facial expressions becomes more ecologically valid. Distal EMG measurements are possible through volume conduction whereby the electrical activity generated by each muscle spreads to adjacent areas [65]. The information picked up by the four channels is then used to approximate different sources for the muscular activity. Since four channels are recorded, four sources can be estimated using Independent Component Analysis (ICA) [87]. EMG measured from the *Corrugator Supercilii* and *Zygomaticus Major* muscles is robust for identifying positive and negative expressions [65]. Moreover, the muscular activity for happy facial expressions is sufficiently large to be robust against non-affective facial movements such as chewing gum and biting [8, 68]. Thus, the separated muscle activity contains components for muscles involved in generating smiles and can be used to identify these and other types of expressions using machine learning. It has been shown that this EMG wearable approach is able to detect both smiles, i.e., positive valence; and frowns, i.e., negative valence [68] using the signal’s magnitude. This is possible even in real time [70], and the approach can also be used offline for fast and subtle spontaneous smile identification [71]. Finally, this device has also been used to analyse spatio-temporal features of a smile by fitting envelopes to the EMG’s Independent Components (ICs), and later performing automatic peak detection on that envelope [72] with similar performance to that achieved by Computer Vision [73].
- **Video recordings**. A video of the producer’s facial expressions was recorded using a Canon Ivis 52 camera at 30 FPS.
- **Self-report questionnaires**. The producers were asked to report how often they smile in everyday situations; the Affect Grid [88] was used as a measure of explicit affect self-report in a dimensional space; and the Implicit Positive and Negative Affect Test (IPANAT) [89, 90] was used as a measure of implicit affect. The Affect Grid is a measure that estimates the affective state of a person by asking explicitly about the degree of valence (ranging from unpleasant to pleasant) and arousal (ranging from sleepiness to high arousal) experienced in a 9-point scale. On the other hand, the IPANAT operates as an implicit measure of affect through an affect misattribution process. Ambiguous stimuli are presented, namely a set of nonsense words, the affective value of which is rated on a six-point scale in relation to 12 emotional adjectives. The assumption is that people respond in accordance with their current affective state, without being fully aware of the construct being measured [91]. Additionally, the producers were asked to rank the videos in order of preference after the spontaneous block and to report if they had seen the videos before or if they would watch them again. At the end of the experiment, control questions were asked about age, gender, hometown, and how often they smiled in everyday life.
- **Self video rating**. The producers tagged the onset and offset of their own facial expressions using Dartfish Version 3.2 software. They labelled each expression as spontaneous or posed and indicated whether or not the expression was a smile.
- **Third person video rating**. In addition to the producers’ own video rating, two independent raters aware of the hypothesis labelled the videos. They used the same software as the producers (Dartfish Version 3.2). They coded for the start frame and the duration of every facial expression. They labelled each expression as a smile or another facial expression, and as a posed or spontaneous expression. Smiles were often a display of AU06 and/or AU12. However, the smile label was not assigned every time these AUs occurred.

### Apparatus

All stimuli were presented to the producers on a Philips B-line 240B4 24-inch monitor with a resolution of 1920 x 1200 pixels. An MSi GP602PE230 laptop was used to present the stimuli. It was connected via a USB to a custom hardware circuit. This circuit received wireless signals from a remote controller used by the experimenter to start the stimuli. Once the stimuli had started, a hardware trigger was sent to the Smile-reader. Finally, another two laptops were used. The first, a Dell Latitude E6230, was used to record the EMG data received from the Biolog device via Bluetooth. The second, a Dell Inspiron N5110, was used to let the producers self video rate their facial expressions.

### Analysis and results

#### Self-report

**Fig 3:**
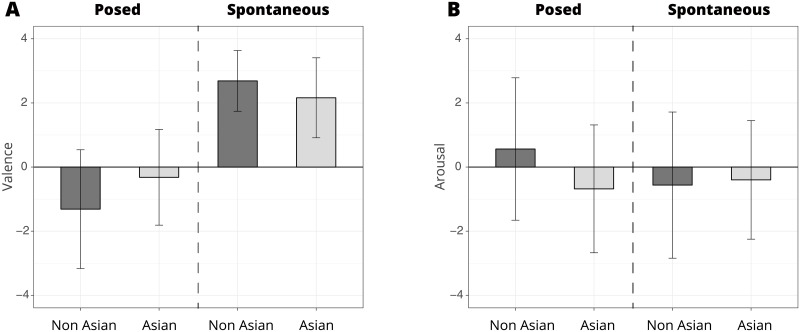
Affect grid ratings for each experimental block and ethnicity. The average valence (A) and arousal (B) ratings for each experimental block and nationality are shown. The producers reported a more positive valence during the spontaneous block, and a less positive valence during the posed block. However, arousal did not differ between blocks.

35 of the 41 producers said they would watch one or more of the shown videos again and 27 had already seen at least one of the stimuli before. A one-factor ANOVA revealed no significant self-perceived differences in how much the producers smiled on a daily basis (Dependent Variable, DV) per ethnicity (Independent Variable, IV) (*F*(1,39) = 1.72, *p* = .20, $\eta_{p}^{2}=0.04$). A 3-factor mixed ANOVA (n = 41, repeated measures experimental block = 2) with the Affect Grid valence as the DV; and the experimental block, ethnicity, gender and their interactions as IVs, yielded significant results only for experimental block (*F*(1,72) = 11.76, *p* = .001, $\eta_{p}^{2}=0.14$). Ethnicity (*F*(1,72) = 0.26, *p* = .61, $\eta_{p}^{2}=0.02$) and gender (*F*(1,72) = 0.49, *p* = .45, $\eta_{p}^{2}=0.01$) were non-significant. The only significant interaction effect was the interaction between ethnicity and block (*F*(1,72) = 4.13, *p* = .045, $\eta_{p}^{2}=0.05$). The interactions between ethnicity and gender (*F*(1,72) = 0.001, *p* = .97, $\eta_{p}^{2}=0.01$), experimental block and gender (*F*(1,72) = 2.07, *p* = .16, $\eta_{p}^{2}=0.02$), and the three-way-interactions were non-significant (*F*(1,72) = 2.244, *p* = .14, $\eta_{p}^{2}=0.03$). Fig 3A shows the differences in valence ratings between the posed and spontaneous experimental blocks, and between different ethnicities. The producers reported more positive feelings during the spontaneous block than during the posed block.

A similar ANOVA using the Affect Grid arousal as the DV, and the experimental block, ethnicity, gender, and their interactions as IVs showed no significant differences in ethnicity (*F*(1,72) = 0.20, *p* = .66, $\eta_{p}^{2}=0.01$), experimental block (*F*(1,72) = 0.77, *p* = .39, $\eta_{p}^{2}=0.01$), gender (*F*(1,72) = 0.43, *p* = .51, $\eta_{p}^{2}=0.01$), the interaction between ethnicity and experimental block (*F*(1,72) = 1.56, *p* = .22, $\eta_{p}^{2}=0.02$), the interaction between ethnicity and gender (*F*(1,72) = 1.40, *p* = .24, $\eta_{p}^{2}=0.02$), the interaction between experimental block and gender (*F*(1,72) = 0.12, *p* = .73, $\eta_{p}^{2}=0.01$), nor the three-way-interactions (*F*(1,72) = 0.11, *p* = .75, $\eta_{p}^{2}=0.01$). The interaction effects were also non-significant. Fig 3B shows the differences between the arousal ratings of the posed and spontaneous experimental blocks, and between different ethnicities.

Analogously, a 3-factor mixed ANOVA with the IPANAT scores (n = 41, repeated measures experimental block = 2 and affect valence = 2) as the DV and experimental block, ethnicity, and reported affect valence (positive or negative) as the IVs yielded significant differences in ethnicity (*F*(1,152) = 7.31, *p* = .008, $\eta_{p}^{2}=0.05$). However, with experimental block (*F*(1,152) = 0.03, *p* = .86, $\eta_{p}^{2}=0.01$), and reported positive or negative affect (*F*(1,152) = 0.04, *p* = .84, $\eta_{p}^{2}=0.01$) the differences were non-significant. Also, none of the interaction effects were significant.

#### Video rating

272 smiles were elicited from 32 producers according to their own video rating. 127 were spontaneous (mean per producer = 3.54, SD = 3.32), and 145 were posed (mean per producer = 3.10, SD = 1.97). Only three people produced sounds that would be catalogued as laughter. According to the producers’ comments during the video rating part of the experiment, three producers found it difficult to know if their own smiles were spontaneous or posed. They mentioned that sometimes a posed smile transformed into a spontaneous one when they thought about the irony of having to smile at the conflicting stimulus images.

According to the external Coder 1, the Duchenne marker, or AU06, appeared in 95% of the spontaneous smiles, whereas it only appeared in 36% of the posed smiles. Similarly, according to Coder 2, AU06 appeared in 92% of the spontaneous smiles, and in 60% of the posed smiles.

When determining whether the producers were smiling or not, the Cohen’s Kappa for agreement between the two independent coders was 0.59. In the same task, the Fleiss’ Kappa between the two coders and the producer’s own video rating was 0.57. However, the agreement diminished when the task was to determine whether the displayed expressions were posed or spontaneous. The agreement between the two independent coders as regards the posed-spontaneous Cohen’s Kappa was 0.30. When also including the producers’ own rating, the Fleiss’ Kappa was 0.13 (**H1**).

#### Electromyography

**Fig 4:**
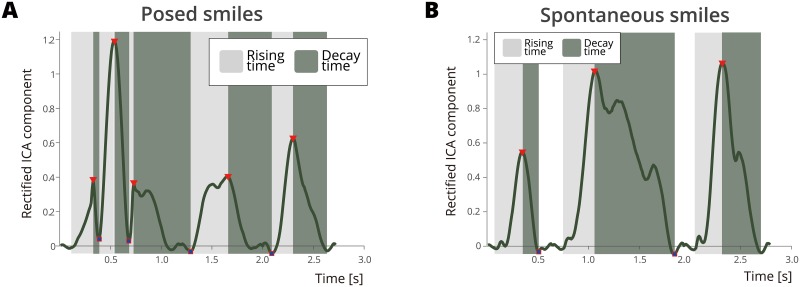
EMG envelopes from posed and spontaneous smiles. A) This shows the processed EMG envelope of self-reported posed smiles during the posed block. This is an example for producer number 37, Independent Component number 1. B) This shows the processed EMG envelope of self-reported spontaneous smiles during the spontaneous block. This is an example for producer 37, Independent Component number 1. The onset and offset speeds and magnitudes of the posed and spontaneous smiles differ significantly.

A similar algorithm to the one described on [72] was used to calculate the temporal features of different smiles. First, the data was band-pass filtered from 5 to 350 Hz and notch filtered at the harmonics of 50 Hz up to 350 Hz [65]. Also, the EMG signal was linearly detrended to prevent signal drifts. Next, the signals from the four channels were decomposed using ICA [87, 92] to separate the distal EMG signals from different source muscles. An envelope was subsequently fitted to the rectified EMG ICs by smoothing the data with an averaging non-overlapping window of 100 ms, and a Savitzky-Golay filter with a 5th order polynomial and a frame length of 41. Then the maximum and minimum points of the envelope were identified. Using the IC peaks as a basis, we calculated the maximum magnitude, onset time, offset time, onset speed, offset speed, and duration and magnitude of the change between a neutral expression and the smile’s apex and used them as features for other analyses (Fig 4).

A series of paired t-tests between posed and spontaneous features obtained from the smiles of the 32 producers who smiled at least once were used to compare posed and spontaneous smile characteristics. Each labelled smile might have had a different number of peaks; therefore, the number of features did not exactly match the number of smiles. The feature data were shuffled randomly and then balanced. The feature vectors of the majority class were under sampled to match the size of the minority class. Afterwards, a series of Bonferroni-corrected t-tests were used to compare the feature vectors of posed and spontaneous smiles. The magnitude of the offset from the smile’s apex and a neutral face was significantly different for posed and spontaneous smiles (*t*(552) = 6.69, *p* < .001, 95% CI [.28 .52], *d* = 0.40), as well as the speed of the offset (*t*(552) = 5.18, *p* < .001, 95% CI [.01 .02], *d* = 0.01). Moreover, the magnitude changes from the smile’s onset until the smile’s apex (*t*(597) = -4.66, *p* < .001, 95% CI [-.40 -.16], *d* = -0.28); onset time (*t*(597) = -2.03, *p* = .043, 95% CI [-7.08 -.123], *d* = -3.96); and onset speed (*t*(597) = -3.13, *p* = .002, 95% CI [-.01 -.003], *d* = -0.01) were significantly different for posed and spontaneous smiles. Table 1 shows the means and standard deviations of the spatio-temporal features calculated for each smile. Furthermore, there were no significant differences between the two ethnicities as regards the production of smiles according to these features.

**Table 1:** EMG spatio-temporal features of spontaneous and posed smiles.

Changes to/from baseline	Spontaneous				Posed			
	Onset		Offset		Onset		Offset	
	Mean	SD	Mean	SD	Mean	SD	Mean	SD
Duration [ms]	37.05*	33.10	44.40	41.05	33.72*	32.74	42.91	44.81
Magnitude change [mV]	1.21*	1.21	-1.22*	1.18	0.89*	0.85	-0.85*	0.79
Speed [mV/ms]	0.05*	0.05	-0.04*	0.05	0.04*	0.04	-0.03*	0.03

Calculated spatio-temporal features. Duration is given in ms. Magnitude of change is derived from the EMG Independent Components. Raw EMG magnitude is given in mV. Speed is given in mV/ms. The results show a difference between spontaneous and posed smiles mainly as regards their temporal dynamics. Asterisks represent the significant differences between posed and spontaneous smiles (*p* < .05).

Moreover, the spatio-temporal features were used to train a Support Vector Machine (SVM), with a radial basis kernel. The goal of using machine learning is twofold. First, it is a test of whether the calculated features, considered together, differ for posed and spontaneous smiles. If the machine is able to correctly classify these features into one class or the other, we can assume that the EMG signatures of the two classes are different. Second, by training a machine to distinguish between the two, we can test H2. If the performance of this artificial intelligence algorithm surpasses that of human judgment, we can assume that human inferential judgements can be enhanced for several applications with the aid of such machines.

To validate the SVM model, a intra-individual cross-validation with 70% training and 15% validation data was used for the 27 producers who displayed at least two posed and two spontaneous smiles. The producers who displayed too few facial expressions for the cross-validation were excluded from this analysis. The results showed that posed and spontaneous smiles could be distinguished with an accuracy of approximately 91% (SD: 4%). Finally, an ANOVA of the performance of the automatic classification for each of the 27 producers as a DV and the ethnicity of the producers as an IV yielded no significant results (*F*(1,25) = 1.37, *p* = .25, $\eta_{p}^{2}=0.05$). Similarly, no gender differences were found (*F*(1,25) = 1.32, *p* = .26, $\eta_{p}^{2}=0.05$).

### Discussion

In this experiment, posed and spontaneous smiles were elicited and analysed. Spontaneous smiles were elicited by showing positively valenced videos to the producers. Posed smiles were requested, even during a slightly unpleasant situation. This experimental design made it possible to control the valence of the affect felt by the producers when they produced the required smiles. As a validation check, self-reported measures of affect were applied, and the producers themselves were asked to label their spontaneous and posed expressions.

From the self-reported measures, a valence difference was observed between spontaneous and posed blocks. As expected, the producers reported feeling more positive during the spontaneous block than during the posed block. This was independent of their ethnicity or gender. On the other hand, no arousal difference was observed between spontaneous and posed blocks. This was probably because of the mildness of the video content. They were pleasant enough for people to smile, but the intensity was similar for the videos of all blocks. As with the arousal, the reported IPANAT scores showed no differences between experimental blocks.

In this experiment, perceptual judgments yielded a higher agreement regarding the existence of a smile than the inferential judgments when distinguishing between posed and spontaneous smiles. Moreover, the agreement between independent coders and the producers’ rating was even lower. These results suggest that, in this case, video rating based solely on visually perceivable cues is not the best way to establish the ground truth. Rather than relying on video rating alone, a good experimental design when collecting the ground truth data is of utmost importance to make inferences about the spontaneity of a smile (**H1**). Furthermore, although the two external coders were experienced coders aware of the hypothesis to be tested, they might not be as good as a certified FACS coder with extensive knowledge on the morphology of posed and spontaneous smiles. It remains to be explored whether expert knowledge would allow coders to accurately distinguish posed from spontaneous smiles.

Previous work has suggested that deliberate smiles are significantly faster and have a higher amplitude than spontaneous smiles [37, 42]. When measured distally, the EMG signatures of the smiles differed in duration and speed, but not magnitude [72]. In this study, the posed smiles were smiles where the intention was to display enjoyment by remembering previously displayed spontaneous smiles. When given this instruction, the two smiles differ significantly in most of the calculated features. The speed of change between a neutral expression and the expression apex of the smiles was significantly different for both onset and offset. Moreover, the onset time, and the magnitude change also differed for both the rising and offset phases. The consistent finding is that the production speed is faster for posed smiles regardless of the elicitation method. However, magnitude differences measured distally seem to depend on the type of smile elicited (**H3**).

Moreover, a high accuracy was achieved by classifying the spatio-temporal features calculated from EMG for the smiles elicited in this study. This further supports the hypothesis that the difference between these smiles can be better detected from their temporal dynamics (**H2**). This is even the case for posed smiles elicited without a temporal constraint enforced by an instruction. Moreover, the Duchenne marker appeared both in spontaneous and posed smiles. This further suggests that the marker can be produced voluntarily [38, 93], and that it should not be considered a marker of spontaneity. This is in line with Ekman’s statement that “muscular activity around the eyes in addition to the smiling lips occurred more often when people were actually enjoying themselves”, the Duchenne marker being present more often but not always [35]. Other more recent studies have suggested that AU6 might signal smile intensity rather than enjoyment in more than one data set [41]. Nevertheless, the difference between genuine or enjoyment smiles and posed ones is faint even for the producers. Three participants mentioned explicitly that “even they found it difficult to know if their own smiles were spontaneous or posed”. Although we discarded the smiles where the participants hesitated for further analyses, this is an interesting observation that points to a mixture between voluntary production and genuine feelings. This idea has already been suggested by the facial feedback hypothesis [94] and in other research considering continuous ratings of genuineness rather than discrete choices [49].

Despite the different ethnicities of the producers involved, no differences were observed regarding the temporal features in their smile EMG results, even for posed smiles. One might expect that people from different places learn different rules of politeness [76–78], or that they smile spontaneously with different frequencies. However, our results did not support such a hypothesis (**H4**).

Finally, a limitation of the analysis is that individual differences were not controlled when calculating the average smile features. The number of features per smile depends on the number of peaks found by the algorithm within one visually tagged event. Smiles are known for their multi-peaked nature, especially during laughter. The obtained features were first randomised, and then a representative, class-balanced sample of feature values could be obtained independently of their producer. Thus, there was no control regarding whether or not the selected features for the t-tests were produced by a small number of producers. Nevertheless, the data used for the SVM was selected from producers that smiled at least twice. Moreover, the trained models were intra-individual. The high discriminability of both types of smiles argues in favour of the feature differences when controlling the feature vectors for specific producers. Therefore, these results should be interpreted carefully, as the means might not represent the exact features that dictate the discriminating power within a participant. Moreover, a limitation of the EMG algorithm is that it requires a certain number of smiles per producer for system calibration. In general, it is difficult to find a one-size-fits-all stimulus to make everybody smile or laugh to achieve such calibration. In our case, despite the fact that producers reported smiling more than 50% of the time, only about 66% of the producers smiled at all with the selected stimuli. Nevertheless, the number of smiles obtained was sufficient to investigate the EMG-based algorithm performance for 27 producers.

## Experiment 2: Human judgment of posed and spontaneous smiles

### Participants

73 volunteers unknown to the producers took part (41 Asian, 37 female, average age = 29 years, SD = 11). Henceforth, the participants in this experiment are referred to as “perceivers”. All the perceivers had normal or corrected to normal vision and provided informed consent before the experiment.

### Stimuli

54 smiles were selected from the smiles gathered in Experiment 1. These were 27 posed smiles and 27 spontaneous smiles from 27 producers (16 Asian; 15 female). The posed smiles were smiles emitted in the posed block, and self-labelled as posed. Similarly, the spontaneous smiles were smiles elicited in the spontaneous block and self-labelled as spontaneous. Smiles of around 5 s were chosen to keep the experiment short. All the stimuli were silent to reduce environmental noise.

### Experimental design and procedure

All the perceivers went through all the selected smiles. A computer program played the stimuli automatically. The perceivers were allowed to watch the stimuli only once. After watching the stimuli, two questions were presented separately. The first was whether they thought the smile was spontaneous or posed, with a forced choice between the two. The second asked how confident they were about their judgment. A Visual Analogue Scale (VAS) was used to report a confidence level of between 0 and 100%. After the perceivers had watched all the stimuli, a structured interview was conducted to request demographic information and inquire about the strategies the perceivers employed to make their judgments.

### Apparatus

All stimuli were presented to the perceiver on an NEC Lavie Hz750/C laptop. The Python toolbox PsychoPy2 version 1.85.4 was used to create an automatic presentation of the stimuli and the subsequent questions.

### Analysis and results

**Fig 5:**
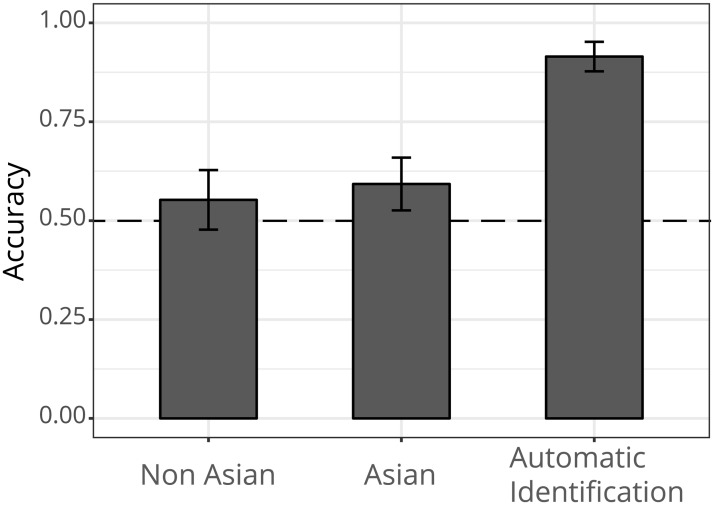
Laypersons’ accuracy when identifying posed and spontaneous smiles. Average accuracy scores are shown. The y-axis represents the accuracy as a percentage. The x-axis shows the three groups. The dotted line represents chance level.

The agreement rate of every perceiver’s posed or spontaneous label with the ground truth label was calculated. Henceforth, this agreement is called accuracy. A one sample t-test showed that the accuracy obtained by the human judges was significantly different from a normal distribution with the mean at chance level (*M* = 0.50, *t*(291) = 7.74, *p* < .001, 95% CI [.56 .59], *d* = 0.57). Fig 5 a plot with the average accuracies for all the groups.

Table 2 shows the number of smiles correctly identified per category. In total, there were 3942 trials (73 perceivers each viewed 54 videos). 2103 posed smile trials were included in the data (53%). There were 1839 spontaneous smile trials (47%). Posed smiles were correctly identified 57% of the time. Analogously, spontaneous smiles were correctly identified 58% of the time. A chi-square test of independence was performed to examine the relationship between stimulus category and the label assigned by the judges. The relationship between these variables was significant, *χ*^2^ (1, *N* = 3942) = 90.52, *p* < .001, which suggests that perceivers are likely to judge posed smiles more accurately than spontaneous smiles. Similar tests were performed for each ethnicity category. The posed smiles were judged more accurately by the two ethnic groups (non-Asians: *χ*^2^ (1, *N* = 1728) = 19.22, *p* < .001. Asians: *χ*^2^ (1, *N* = 1134) = 77.156, *p* < .01).

**Table 2:** Confusion matrix for human judgment.

		Judgement	
		Incorrect	Correct
**Stimuli**	Posed smile	902 (23%)	**1201 (30%)**
	Spontaneous smile	770 (20%)	**1069 (27%)**

This table shows the number of smiles correctly identified by perceivers per category. In total, there were 3942 trials, namely each of the 73 perceivers watched 54 videos. The numbers in parentheses show percentages. Posed smiles were correctly identified 57% of the time, and spontaneous smiles 58% of the time.

Stimuli were annotated regarding whether or not the producer was Asian. This was to assess cross-ethnicity effects when judging the smiles. Then, a match was identified between the ethnicities of the producers and the perceivers. A contingency table was created by comparing ethnic match and mismatch that showed whether or not the perceivers assessed the nature of the producers’ smile correctly (Table 3). There was an ethnic mismatch in 1926 trials (49%), while there was an ethnic match in 2016 of the trials (51%). The judgment was correct 59% of the time when there was an ethnic match, and 56% of the time for ethnic mismatches. The relationship between these variables was non-significant (*χ*^2^ (1, *N* = 3942) = 3.16, *p* = .08), suggesting that perceiver accuracy is independent of the ethnic background of the producer.

**Table 3:** Confusion matrix for cross-ethnicity effect.

		Judgment	
		Incorrect	Correct
**Cross-ethnicity**	Mismatch	845 (22%)	**1081 (27%)**
	Match	827 (21%)	**1189 (30%)**

A match was identified between the ethnicities of the producers and the perceivers. A contingency table was created by comparing ethnic matches and mismatches to whether the perceivers assessed correctly or not the nature of the producer’s smile. In total, there were 3942 trials. The number in parentheses shows percentages. The judgment was correct 59% of the time for ethnic matches, and 56% of the time for ethnic mismatches.

Similar to the procedure described in [48], a generalized mixed linear model was used to analyse the tendencies to answer posed or spontaneous in relation to the expression type and ethnicity of the perceiver. Since there were two smiles per producer in the stimuli, a cross-effect was added to explore the influence of each producer in this judgment. The DV was the perceiver-assigned label. The IVs were the ground-truth expression type label and ethnicity of the perceiver. Additionally, cross-random effects of both the producer and the perceiver were included. The estimated effect of each producer on the perceiver’s judgment was weak (*Beta* = 0.53, 95% CI [.39 .72]). The tendency to respond posed or spontaneous was negligible (*Beta* = 0.09, 95% CI [-.16 .34]). The effect of the type of expression was strong (*Beta* = -0.30, 95% CI [-.44 -.17]), confirming that perceivers tended to correctly label posed smiles more often than spontaneous smiles. The effect of perceiver ethnicity was also negligible (*Beta* = 0.07, 95% CI [-.10 .25]). The interaction between type of expression and the ethnicity of the perceiver was weak (*Beta* = -0.26, 95% CI [-.45 -0.07]).

Although the ability to voluntarily imitate their spontaneous smiles differed between producers, the effect of the producer on each perceiver’s judgment was weak. Moreover, the discrimination accuracy of each perceiver tended to be slightly higher for posed smiles than for spontaneous smiles. Finally, to explore the effects of fatigue, the judgment trials were divided by half, and the accuracy differences between the first and the second half were assessed with a generalized mixed linear model. The difference is negligible (*Beta* = 0.03, 95% CI [-.05 0.11]).

Additionally, the perceivers provided a verbal report regarding the features on which they based their decisions concerning posed and spontaneous smiles. Their responses were transcribed and analysed using affinity diagrams [95]. Eight different features were found. 51 perceivers (70%) mentioned eye movement, referring to the shape of the eyes, whether the producers of the smiles were gazing at the screen, or if they were looking lost. 34 perceivers (47%) also looked at body movements such as body vibration, shrugging, head movement, hiding the face, and the degree of relaxation of the posture of the person smiling. The next most popular feature was mouth shape and movement, mentioned by 29 perceivers (40%). Perceivers looked at the opening and closing of the mouth, and whether or not the smiling person was showing her teeth. The timing of the smile was mentioned by 18 perceivers (25%). This category grouped commentaries describing sudden changes, the duration of the smile, and the simultaneity of eye and mouth movements. Furthermore, 13 perceivers (18%) looked at the intensity of the smiles. With respect to intensity, most perceivers mentioned how wide the smiling person was opening her mouth. Other less popular categories included eyebrow lifts (3 perceivers, 4%), and surprisingly, the beauty of the smile (2 perceivers, 3%). Pearson correlation coefficients were calculated between the participant’s accuracy and feature usage. Only mouth shape movement (*r* = 0.29, *p* = 0.01) and eyebrow lifts (*r* = -0.24, *p* = 0.04) were significantly correlated to accuracy.

### Discussion

This experiment explored human performance when judging between posed and spontaneous smiles. The results suggest that relying only on visible behavioural cues is not as efficient as relying on the spatio-temporal dynamics of electrophysiological cues. Even though the perceiver’s accuracy scores were significantly higher than chance level, their accuracy was significantly lower than that obtained with automatic identification.

Contrary to the veracity effect [96], we found that perceivers tend to make more accurate judgments with posed smiles, despite the negligible bias towards responding more often to posed or spontaneous smiles. It seems that perceivers are more likely to accept that a smile is posed, than accept that it is spontaneous. This might be because of the experimental context in which the producers’ smiles were recorded. Perceivers might have thought that people would not be spontaneous in such a situation.

Moreover, these results were independent of the ethnicity of both the judge and the producer, thus exhibiting no Cross-Ethnicity Effect. Since most producers were Asians, poorer non-Asian accuracy was expected. However, in this experiment, this effect was only a non-significant trend. When comparing the frequencies of correct answers across ethnicity matches and mismatches, an ethnicity mismatch resulted in a moderate decrease in accuracy. Thus, we found no strong evidence supporting the hypothesis that spontaneity judgement accuracy is affected by the ethnicity of the producers and perceivers (**H4a, H4b**). Further research should consider to increase the sample size to increase confidence on these results.

According to the affinity diagram results, the participants used eight features to distinguish between posed and spontaneous smiles. Eye, mouth, and body movement were the most commonly used features. Also, perceivers often reported having looked at the dynamics of the smiles in gaze, mouth, and body movements. From these, only the mouth movement was positively correlated with accuracy, whereas eyebrow movement was negatively correlated. Nevertheless, these correlations were weak. Moreover, context appears to be really important to the human judgment of different smiles. Many perceivers mentioned having tried to guess the context in which the producers were smiling and particularly what they were looking at. Several perceivers mentioned that a producer suddenly smiling with idle gaze constituted posing.

Although the perceivers were not experts in reading posed and spontaneous smiles, they chose appropriate features when making their decisions. However, the results from the aforementioned algorithm were consistently better than human judgment of the same data. Therefore, it seems inappropriate to use “third person rating” as inferential ground truth on the genuineness of a smile (**H1**). Nevertheless, it is important to mention that the participants of study 2 were completely naive, and that experts might have performed better.

All in all, in a context-less environment, automatic identification outperformed a layperson’s judgment based on visible behavioural cues only. Even though the perceivers were not experts in reading posed and spontaneous smiles, they chose appropriate features when making their decisions. From the eight features mentioned, the most common are relevant discrimination features according to the literature [17, 97]. Nevertheless, it is difficult to distinguish posed and spontaneous smiles from their spatial visible cues alone as their dynamics play an important role.

A limitation of this study is the reduced amount of information available for the perceivers to make their choices. The provided stimuli were stripped from many contextual and multi-sensory cues that might have proven useful for the human perceivers. Since a human speciality is the ability to integrate information from multimodal channels, these results might not have favoured them in terms of making relevant decisions. It could be that they were so focused on trying to guess the context that they missed other important available visual cues. Moreover, the 50-50 forced choice might have limited the ecological validity of experiment 2 by capitalizing on cultural expectations of the signalling behaviour according to each perceiver’s definition of posed and spontaneous. Future work should explore potential differences in performance when the answer is not restricted to two options. For example, [49] argued that the genuineness of a facial display is not categorical, but rather continuous to allow for mixtures of genuineness and politeness. Hence, it was suggested that perceived genuineness be rated using a neutral-midpoint scale ranging from completely fake (-7) to completely genuine (+7). This would also make it possible to gather data on both relative and absolute perceptions. Furthermore, placing the smile reader next to the eyes of the producers might have been distracting, along with possible occlusion caused by the fact that some producers wore glasses. If the Duchenne marker is indeed an important clue to the spontaneity of human judgments, these factors might have hindered the perceiver’s performance. Finally, experiment 2 was somewhat long. Although we found out that there are no performance differences between the first and the second half of the experiment, fatigue might have hindered the performance of the perceivers.

## Conclusion and future work

Facial expressions are an important factor when assessing affective responses. Although they usually correlate with subjective affective experiences, they can also be controlled voluntarily to convey a message. Therefore, we must understand the differences between posed and spontaneous facial expressions if we are to assess affective reactions. In this paper, we investigated the differences between both types of smiles using facial distal EMG. Posed and spontaneous smiles were found to differ in magnitude and speed for onset and offset. These features appear to be more robust than visual markers such as the Duchenne marker. This marker also appeared in posed smiles despite the belief that it is an indicator of spontaneity. Moreover, laypersons can distinguish between posed and spontaneous smiles at above chance level by using relevant features. However, their accuracy is not very high. Whereas there was good perceptual agreement between a smile producer and a third person coder regarding the existence of a smile, the agreement worsened considerably for inferences about smile spontaneity. This suggests that third person’s view on the meaning of a smile appears to be inaccurate (**H1**). However, it remains to be investigated whether certified FACS coders with expert knowledge on psychological theories of the coherence between facial expressions and emotion would assess the facial expressions more accurately than a layperson.

On the other hand, the automatic identification of posed and spontaneous smiles achieved an accuracy of 94%. This might be because of the high temporal resolution of EMG measurements, which makes it suitable for observing dynamic features critical to this task. Therefore, automatic detection using electrophysiological signals appears to be advantageous in this scenario (**H2**). Although this might partially be because these cues are not directly perceivable by humans [73], other methods such as CV-based automatic algorithms might lead to similar conclusions. Nevertheless, our results suggest that automatic identification tools have the potential to complement the human ability to interpret information contextually. Although in this occasion we have chosen EMG-based detection for practical reasons, we expect that FACS analysis, either expert driven, automated or a hybrid of both, could yield similar results with less obtrusiveness. Future work should explore smiles produced in this experimental setup with vision-based methods for more detailed insights on AU production and perception. Moreover, it is left for future work to analyse the synchronization of EMG signals between producers and perceivers and how these are related to perceiver’s accuracy.

As in previous studies, the relevant features for this discrimination appear to be the onset and offset speeds of change and onset duration. However, the results of experiment 1 indicated that magnitude features also become relevant when posed smiles are intended to convey enjoyment and their duration is not constrained by an instruction (**H3**).

In this study, no ethnic differences were found as regards smile production. However, a trend indicating cross-ethnicity effects was found when assessing the ethnic match between producers and perceivers (**H4**). A recent study has suggested that a smile is easier to interpret in heterogenous societies, and that cultures with ancestral diversity tend to smile more often [75]. This is in line with our observation that three participants were smiling spontaneously even in the presence of negative stimuli. Nevertheless, our sample size was too limited to allow us to reach a clear conclusion about cultural differences. Future work should explore whether our results can be generalised.

All in all, we believe that, in the future, the automatic identification of positive affective states will be helpful in terms of further assessing the relationship among emotion expression, emotion experience, and emotion recognition. Understanding the relationship between these domains of emotion will help to shed light on the mechanisms of our affective experiences and infer their psychological significance. A wearable such as the smile-reader could be used to develop better tools for assessing well-being and treatments for both healthy perceivers and people with psychological disorders. For example, people with schizophrenia have been reported to exhibit a flat affect expression despite being able to experience and report intense emotions [98–100]. This effect might be due to demand characteristics. However, some evidence has shown that they exhibit similar skin conductance responses during affective episodes as controls [98]. Hence, by measuring electrophysiological signals and self-report simultaneously, we could be able to gather more clues to the relationship between embodied responses and subjective experience.
